# NFE2L2 Is a Potential Prognostic Biomarker and Is Correlated with Immune Infiltration in Brain Lower Grade Glioma: A Pan-Cancer Analysis

**DOI:** 10.1155/2020/3580719

**Published:** 2020-10-09

**Authors:** Qiang Ju, Xinmei Li, Heng Zhang, Songxia Yan, Ying Li, Yanjie Zhao

**Affiliations:** ^1^Department of Blood Transfusion, The Affiliated Hospital of Qingdao University, Qingdao University, Qingdao, China; ^2^School of Public Health, Qingdao University, Qingdao, China

## Abstract

Nuclear factor, erythroid 2 like 2 (NFE2L2, NRF2) is a transcription factor that regulates various antioxidant enzymes. It plays a vital physiological role in regulating oxidative stress and inflammatory response. However, the roles of NFE2L2 in human cancers are still unclear. Our study is aimed at analyzing the prognostic value of NFE2L2 in pan-cancer and at revealing the relationship between NFE2L2 expression and tumor immunity. The present study revealed that NFE2L2 was abnormally expressed and significantly correlated with mismatch repair (MMR) gene mutation levels and DNA methyltransferase expression in human pan-cancer. In particular, pan-cancer survival analysis indicated that NFE2L2 expression was associated with adverse outcomes—overall survival (OS), disease-specific survival (DSS), and progression-free interval (PFI)—in adrenocortical carcinoma (ACC), brain lower grade glioma (LGG), and pancreatic adenocarcinoma (PAAD) patients. A positive relationship was also found between NFE2L2 expression and immune infiltration, including B cells, CD4+ T cells, CD8+ T cells, neutrophils, macrophages, and dendritic cells, especially in breast invasive carcinoma (BRCA), colon adenocarcinoma (COAD), kidney renal clear cell carcinoma (KIRC), LGG, liver hepatocellular carcinoma (LIHC), and prostate adenocarcinoma (PRAD). Additionally, NFE2L2 expression was positively correlated with the immune score and the expression of immune checkpoint markers in LGG. In conclusion, these results indicate that transcription factor NFE2L2 is a potential prognostic biomarker and is correlated with immune infiltration in LGG.

## 1. Introduction

Nuclear factor, erythroid 2 like 2 (NFE2L2) is a redox-sensitive transcription factor localized mainly in the cytoplasm. It is ubiquitously expressed in the esophagus, thyroid, and other tissues [1, 2]. NFE2L2-mediated oxidative stress is a prominent feature of cervical cancer [3], promoting the proliferation, inhibiting the apoptosis, and enhancing the migration and invasion of cervical cancer cells [4, 5], as well as increasing the tumor chemoresistance [6, 7], suggesting that NFE2L2 may be a marker of poor prognosis in cervical cancer patients [8]. In addition, the redox subtype of lung squamous cell carcinoma (LUSC) is driven by genomic mutations in the NFE2L2/KEAP1 complex [9]. Although cervical cancer and LUSC have been studied, many research gaps remain across the cancer spectrum.

The tumor microenvironment (TME) is very complex and contains both the cellular and noncellular components. On the one hand, inflammatory cells, including neutrophils and myeloid-derived suppressor cells (MDSCs), suppress beneficial immune functions in the TME, preventing normal immune cells from attacking tumor cells and promoting tumor growth [10, 11]. On the other hand, immune cell infiltration of the TME constitutes a strategy used by tumor cells to evade immune-mediated killing [12–14]. Tumor-associated macrophages (TAMs) mediate immune escape and then play important roles in tumorigenesis and development [15–18]. Currently, immunotherapy is a trending topic in tumor therapy. For example, programmed death-1 (PD-1) and programmed death-ligand-1 (PD-L1) inhibitors have been found to induce tumor cell apoptosis by blocking the PD-1/PD-L1 signaling pathway, thus playing an effective antitumor role in lung cancer and melanoma [13, 19–21]. However, the role and underlying mechanisms of NFE2L2 in tumor immunity are unknown.

In the current study, we comprehensively analyzed the association between NFE2L2 expression and patients' prognosis in 33 cancer types. In addition, we explored the correlation between NFE2L2 expression and tumor immunity. Our findings revealed the possible role of NFE2L2 across cancers, suggesting that NFE2L2 is a potential prognostic biomarker and is correlated with immune infiltration in many cancers, especially in LGG.

## 2. Materials and Methods

### 2.1. Sample Information and NFE2L2 Expression Analysis in Human Pan-Cancer

NFE2L2 gene expression data in 31 normal tissues and 21 tumor cell lines were obtained from the Genotype-Tissue Expression (GTEx) portal (https://gtexport.org/home/) and Cancer Cell Line Encyclopedia (CCLE) database (https://portals.broadinstitute.org/ccle/about). The difference in NFE2L2 expression between cancer and normal tissues was analyzed by combining the data for normal tissues from the GTEx database with the data from The Cancer Genome Atlas (TCGA). Level 3 RNA sequencing data and clinical follow-up information for patients with 33 types of cancers (ACC: adrenocortical carcinoma; BLCA: bladder urothelial carcinoma; BRCA: breast invasive carcinoma; CESC: cervical squamous cell carcinoma; CHOL: cholangiocarcinoma; COAD: colon adenocarcinoma; DLBC: lymphoid neoplasm diffuse large B cell lymphoma; ESCA: esophageal carcinoma; GBM: glioblastoma multiforme; LGG: brain lower grade glioma; HNSC: head and neck squamous cell carcinoma; KICH: kidney chromophobe; KIRC: kidney renal clear cell carcinoma; KIRP: kidney renal papillary cell carcinoma; LAML: acute myeloid leukemia; LIHC: liver hepatocellular carcinoma; LUAD: lung adenocarcinoma; LUSC: lung squamous cell carcinoma; MESO: mesothelioma; OV: ovarian serous cystadenocarcinoma; PAAD: pancreatic adenocarcinoma; PCPG: pheochromocytoma and paraganglioma; PRAD: prostate adenocarcinoma; READ: rectum adenocarcinoma; SARC: sarcoma; SKCM: skin cutaneous melanoma; STAD: stomach adenocarcinoma; TGCT: testicular germ cell tumors; THCA: thyroid carcinoma; THYM: thymoma; UCEC: uterine corpus endometrial carcinoma; UCS: uterine carcinosarcoma; and UVM: uveal melanoma) were obtained from TCGA database. All expression data were normalized through log2 conversion.

### 2.2. MMR Gene Mutation and DNA Methyltransferase Analysis

Abnormalities in the DNA mismatch repair system (MMRs) can lead to tumorigenesis [22]. The mutation levels of 5 MMR genes (MLH1, MSH2, MSH6, PMS2, and EPCAM) were obtained from TCGA database. Pearson correlation analysis was used to evaluate the relationship between NFE2L2 expression and MMR gene mutation levels. In addition, DNA methyltransferases play an important role in altering chromatin structure and gene expression [23]. The relationship between the expression level of NFE2L2 and that of 4 methyltransferases (DNMT1, DNMT2, DNMT3A, and DNMT3B) was evaluated by Pearson correlation analysis.

### 2.3. Survival and Prognosis Analysis

The relationship between NFE2L2 gene expression and patients' prognosis (OS: overall survival; DSS: disease-specific survival; DFI: disease-free interval; and PFI: progression-free interval) in 33 cancers was visualized with forest plots and Kaplan-Meier curves. The hazard ratio (HR) and 95% confidence intervals were calculated via univariate survival analysis.

### 2.4. Correlations between NFE2L2 Expression and Immune Characteristics in the TIMER Database

The Tumor Immune Estimation Resource (TIMER) database contains 10,897 samples from TCGA (https://cistrome.shinyapps.io/timer/). RNA-seq expression profile data were used to evaluate the infiltration of 6 immune cells (B cells, CD4+ T cells, CD8+ T cells, neutrophils, macrophages, and dendritic cells) in tumor tissues. The scores of these 6 infiltrating immune cells in 33 cancers were downloaded from the TIMER database. Spearman correlation analysis was used to evaluate the correlation between NFE2L2 expression and immune infiltration. In addition, we evaluated the relationship between NFE2L2 expression and the immune/stromal scores (ImmuneScore and StromalScore) and immune checkpoint marker expression levels by Spearman and Pearson correlation analyses, respectively. Gene expression levels are shown as log2 RSEM values.

### 2.5. Statistical Analysis

The Kruskal-Wallis test was adopted to analyze NFE2L2 expression levels in different tissues and cancer cell lines. Differences in NFE2L2 expression levels in tumor tissues and normal tissues were evaluated by a *t*-test. In survival analysis, the HRs and *P* value were calculated by univariate Cox regression analysis. Kaplan-Meier curves were used to compare the survival of patients stratified according to different levels of NFE2L2 expression. *P* < 0.05 was set as the significance threshold for all statistical analyses.

## 3. Results

### 3.1. The mRNA Expression Level of NFE2L2 in Human Pan-Cancer

**Figure 1:**
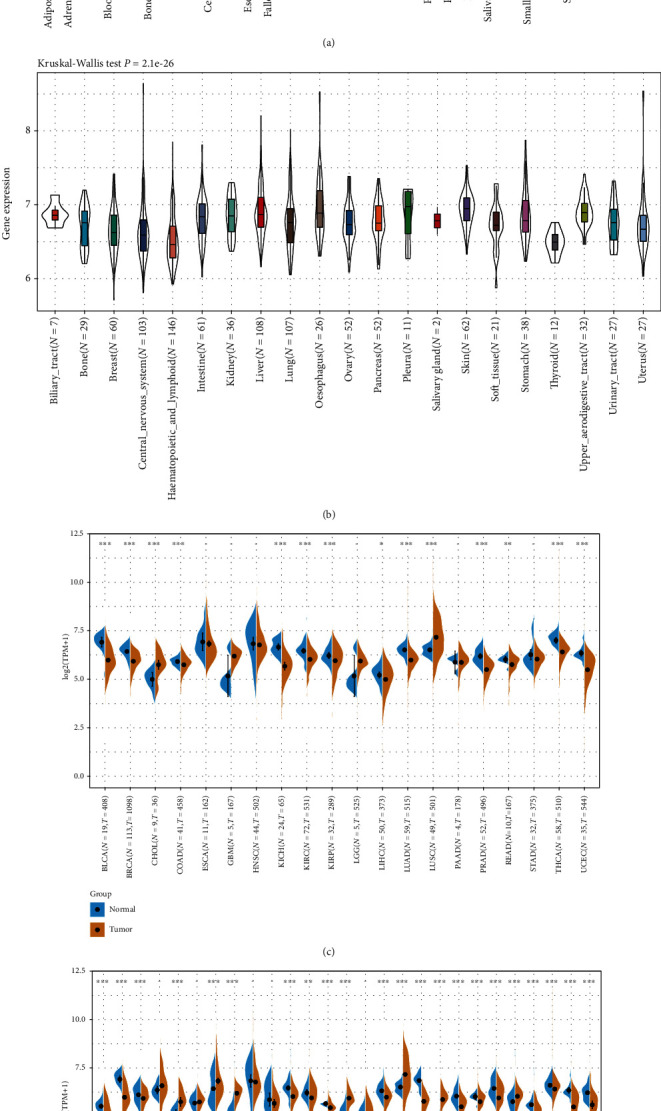
NFE2L2 is abnormally expressed in pan-cancer. (a) NFE2L2 expression in 31 normal tissues from the GTEx database. (b) NFE2L2 expression in 21 tumor cells from the CCLE database. (c) Differential expression of NFE2L2 in cancers and normal tissues from TCGA database. (d) NFE2L2 expression in 27 cancer types from the GTEx database and TCGA database (^∗^*P* < 0.05, ^∗∗^*P* < 0.01, and ^∗∗∗^*P* < 0.001).

First, we analyzed NFE2L2 expression in 31 types of tissues using the GTEx dataset. As shown in Figure 1(a), NFE2L2 was generally highly expressed in the bladder, ovary, vagina, and thyroid tissues. Furthermore, we downloaded the data of tumor cell lines from the CCLE database and analyzed NFE2L2 expression in these tumor cell lines. Results showed that NFE2L2 was expressed in all 21 kinds of tumor cell lines (Figure 1(b)). To further determine the differences in NFE2L2 expression between the tumor and normal tissues, we obtained NFE2L2 expression data from TCGA database. As shown in Figure 1(c), NFE2L2 expression was significantly higher in CHOL and LUSC tissues than in normal tissues. However, it was significantly lower in BLCA, BRCA, COAD, KICH, KIRC, KIRP, LIHC, LUAD, PRAD, READ, THCA, and UCEC compared with normal tissues. Due to the small number of normal tissue samples in TCGA database, we further integrated the normal tissue data from the GTEx database and the tumor tissue data from TCGA database to analyze the differences in NFE2L2 expression in 27 cancer types. Results revealed that NFE2L2 was abnormally expressed in 22 of these cancers. Specifically, NFE2L2 expression was higher in tissues from 7 cancers (CHOL, ESCA, GBM, LGG, LUSC, PAAD, and STAD) and lower in tissues from 15 cancers (ACC, BLCA, BRCA, KIRC, KIRP, LAML, LUAD, OV, PRAD, READ, SKCM, TGCT, THCA, UCEC, and UCS) than in the normal tissues (Figure 1(d)). Taken together, these results reveal that NFE2L2 is abnormally expressed in different cancers.

### 3.2. NFE2L2 Is Correlated with MMR Gene Mutation Levels and DNA Methyltransferase Gene Expression in Human Pan-Cancer

**Figure 2:**
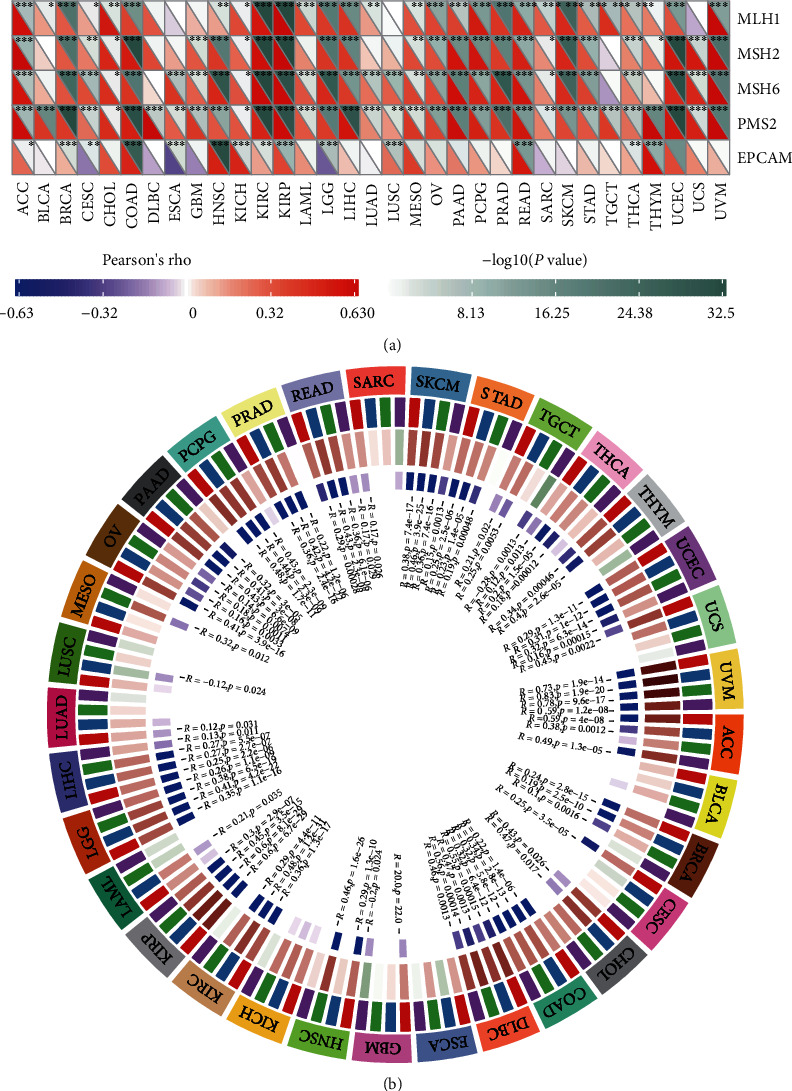
NFE2L2 expression is correlated with MMR gene mutation levels and DNA methyltransferase expression in pan-cancer. (a) Pearson correlation analysis of NFE2L2 expression with the mutation levels of 5 MMR genes (MLH1, MSH2, MSH6, PMS2, and EPCAM) in pan-cancer (^∗^*P* < 0.05, ^∗∗^*P* < 0.01, and ^∗∗∗^*P* < 0.001). (b) Pearson correlation analysis of NFE2L2 expression with that of 4 DNA methyltransferases (DNMT1, DNMT2, DNMT3A, and DNMT3B) in pan-cancer.

MMRs is a DNA damage repair mechanism. Functional loss of key genes in this mechanism leads to DNA replication errors [24], higher somatic mutations, and tumorigenesis [22, 25]. To evaluate the role of NFE2L2 in tumorigenesis, we analyzed the correlation between NFE2L2 expression and MMR gene mutation levels. Results showed that NFE2L2 expression was positively related to the mutation levels of 5 MMR genes (MLH1, MSH2, MSH6, PMS2, and EPCAM) in human cancers (Figure 2(a)).

DNA methylation is an epigenetic modification that can alter gene expression [26]. Alteration of the DNA methylation status is an important factor in tumorigenesis [27]. Next, we further evaluated the correlation between NFE2L2 expression and that of 4 DNA methyltransferases. Evidently, NFE2L2 expression is closely related to the expression of DNMT1, DNMT2, DNMT3A, and DNMT3B across human cancers, especially in COAD, KIRP, LGG, and UVM (Figure 2(b)). In summary, these results indicate that NFE2L2 may mediate tumorigenesis by regulating DNA damage or methylation.

### 3.3. Prognostic Value of NFE2L2 in Human Pan-Cancer

**Figure 3:**
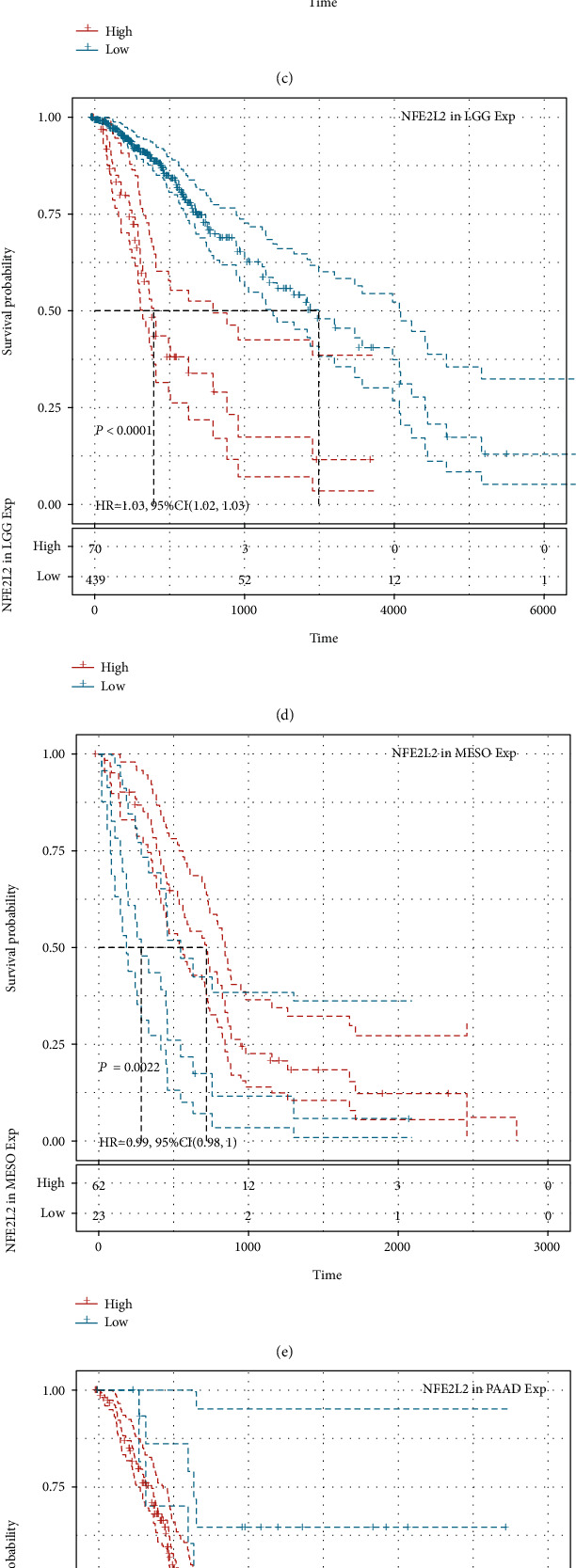
Relationship of NFE2L2 expression with patients' OS. (a) Forest plots showing the HRs related to NFE2L2 expression in 33 cancer types. (b–h) Kaplan-Meier OS curves for patients stratified by different expression levels of NFE2L2 in 7 cancer types.

**Figure 4:**
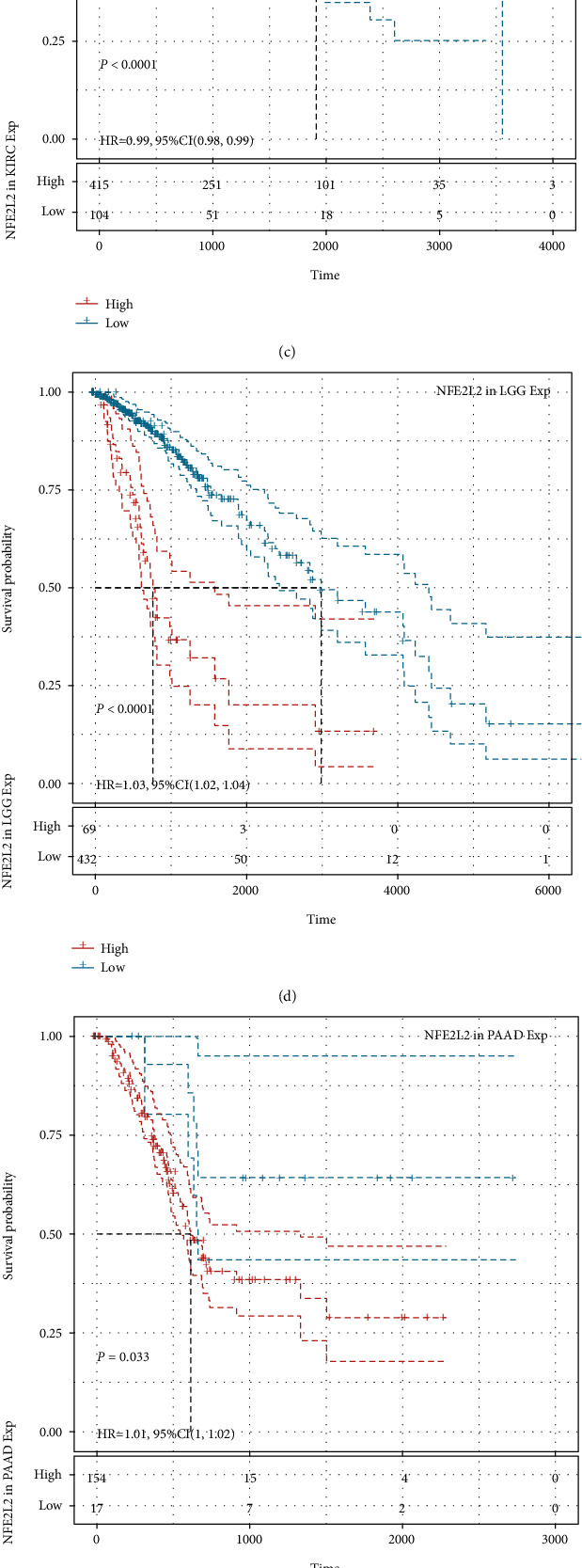
Relationship of NFE2L2 expression with patients' DSS. (a) Forest plots showing the HRs related to NFE2L2 expression in 33 cancer types. (b–g) Kaplan-Meier DSS curves for patients stratified by different expression levels of NFE2L2 in 6 cancer types.

**Figure 5:**
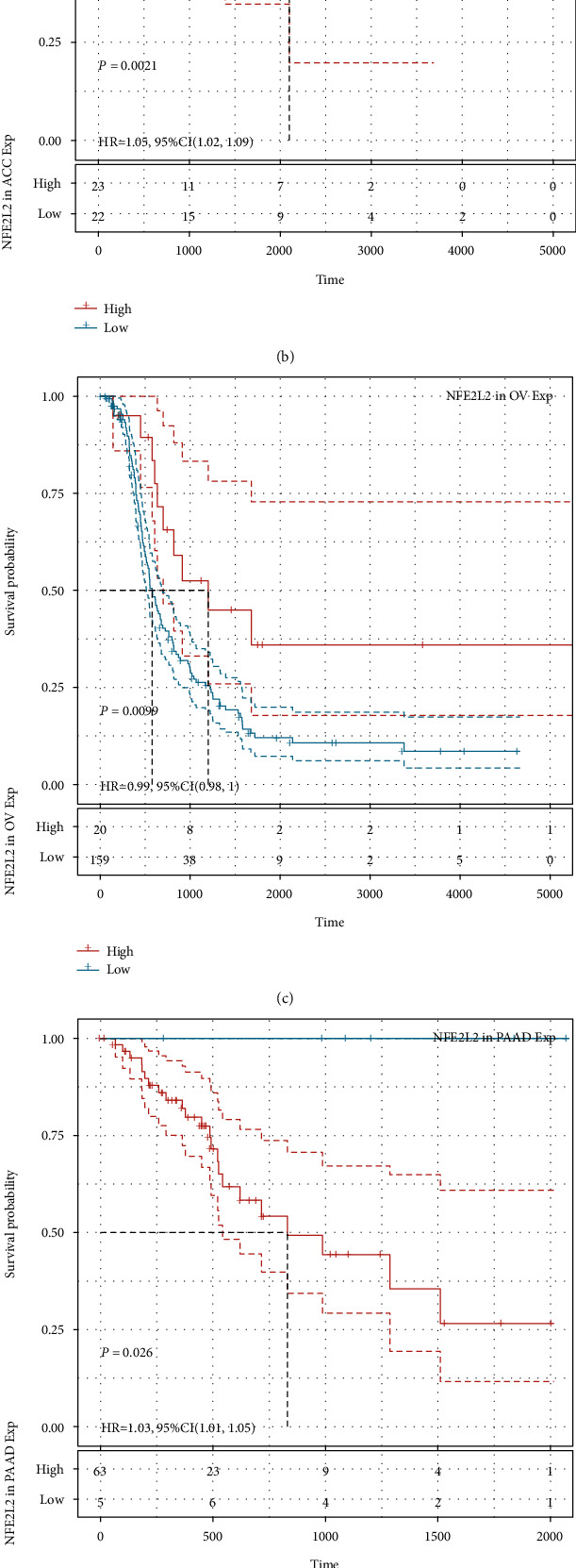
Relationship of NFE2L2 expression with patients' DFI. (a) Forest plots showing the HRs related to NFE2L2 expression in 33 cancer types. (b–e) Kaplan-Meier DFI curves for patients stratified by different expression levels of NFE2L2 in ACC, OV, PAAD, and PRAD.

**Figure 6:**
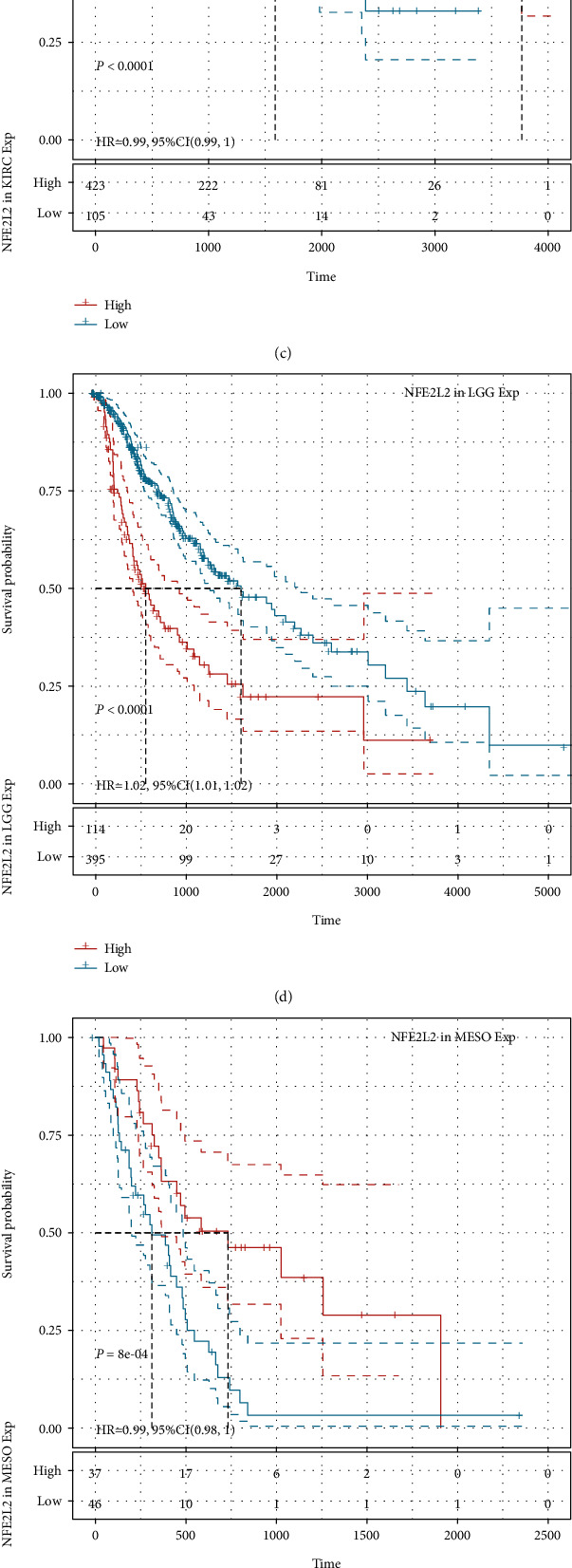
Relationship of NFE2L2 expression with patients' PFI. (a) Forest plots showing the HRs related to NFE2L2 expression in 33 cancer types. (b–g) Kaplan-Meier PFI curves for patients stratified by different expression levels of NFE2L2 in 6 cancer types.

Next, we investigated the relationship between NFE2L2 expression and the prognosis of patients in pan-cancer. Notably, NFE2L2 expression was significantly correlated with patients' OS in 7 types of cancer (ACC, KIRC, LGG, MESO, PAAD, SARC, and UCS) (Figure 3(a)). Specifically, NFE2L2 appeared to be a risk factor in 4 cancer types: ACC (*P* = 0.0016, HR = 1.03), LGG (*P* < 0.0001, HR = 1.03), PAAD (*P* = 0.0076, HR = 1.01), and UCS (*P* = 0.00019, HR = 1.02). In addition, NFE2L2 was a protective factor in 3 other types of cancer: KIRC (*P* < 0.0001, HR = 0.99), MESO (*P* = 0.0022, HR = 0.99), and SARC (*P* = 0.0033, HR = 0.99) (Figures 3(b)–3(h)). Since non-tumor-related factors may cause death during follow-up, we then analyzed the relationship between NFE2L2 expression and DSS in 33 cancers. Results showed NFE2L2 expression impacted patients' DSS in 6 cancer types (ACC, KIRC, LGG, PAAD, SARC, and UCS) (Figure 4(a)). Specifically, Kaplan-Meier curves showed that high expression of NFE2L2 was significantly correlated with poor prognosis of patients in ACC (*P* = 0.015, HR = 1.02), LGG (*P* < 0.0001, HR = 1.03), PAAD (*P* = 0.033, HR = 1.01), and UCS (*P* = 0.00038, HR = 1.01) and reversely in KIRC (*P* < 0.0001, HR = 0.99) and SARC (*P* = 0.014, HR = 0.99) (Figures 4(b)–4(g)). Subsequently, we investigated the relationship between NFE2L2 expression and DFI and found that increased NFE2L2 expression was correlated with poor prognosis in ACC (*P* = 0.0021, HR = 1.05) and PAAD (*P* = 0.026, HR = 1.03) but with favorable prognosis in OV (*P* = 0.0099, HR = 0.99) and PRAD (*P* = 0.00044, HR = 0.98) (Figure 5). Moreover, we assessed the relationship between NFE2L2 expression and PFI. The results showed that high expression of NFE2L2 affected PFI unfavorably in ACC (*P* < 0.0001, HR = 1.03), LGG (*P* < 0.0001, HR = 1.02), PAAD (*P* = 0.013, HR = 1.01), and UVM (*P* = 0.0062, HR = 1.03) but favorably in KIRC (*P* < 0.0001, HR = 0.99) and MESO (*P* = 8*e* − 04, HR = 0.99) (Figure 6). In conclusion, these results suggest that NFE2L2 expression is significantly correlated with the prognosis of patients, especially those with ACC, LGG, and PAAD.

### 3.4. NFE2L2 Expression Is Correlated with Immune Infiltration Levels and Immune Checkpoint Marker Expression across Cancers

**Figure 7:**
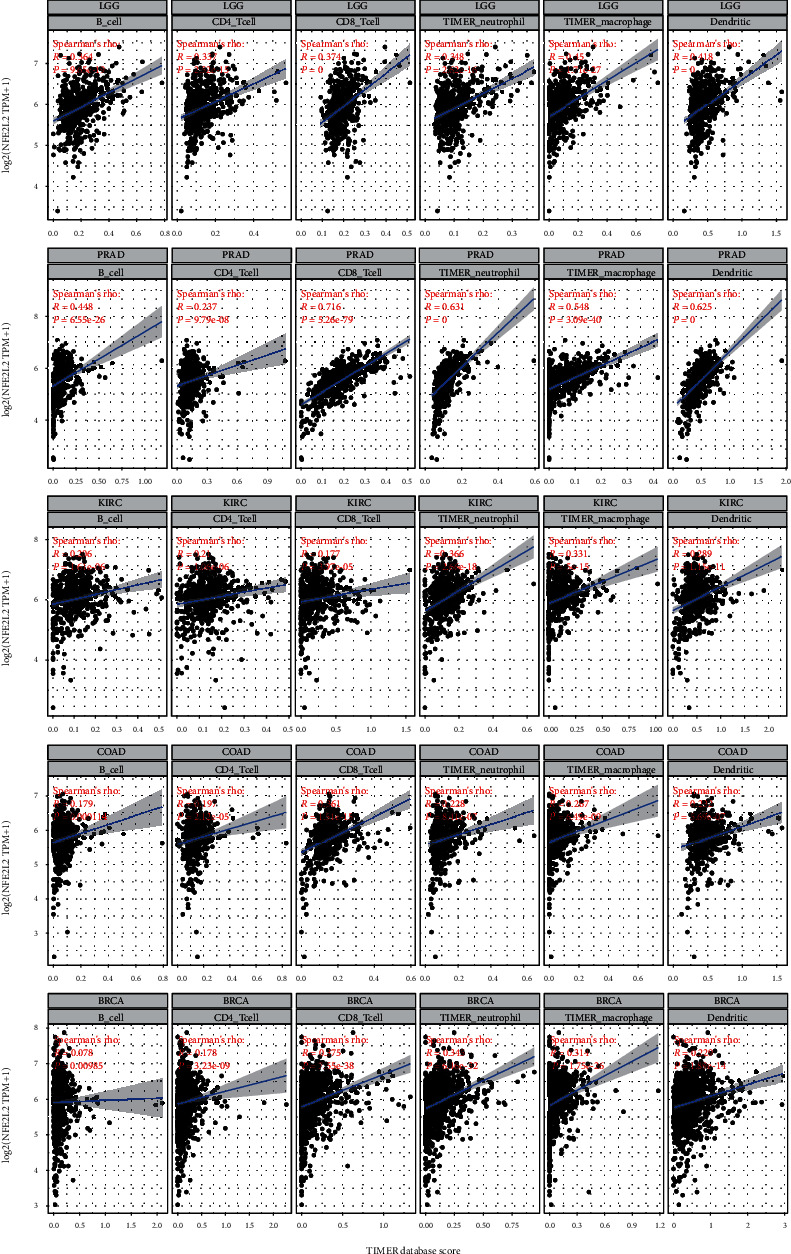
Correlation of NFE2L2 expression with immune infiltration levels of B cells, CD4+ T cells, CD8+ T cells, neutrophil cells, macrophage cells, and dendritic cells.

Immune cells in the TME affect patients' survival [28]. Therefore, the correlation between NFE2L2 expression and immune infiltration in human pan-cancer was further studied. First, we downloaded the scores of 6 types of infiltrating immune cells in 33 types of cancer from the TIMER database and then analyzed the correlation between the NFE2L2 expression level and immune infiltration levels. Results showed that NFE2L2 expression was appreciably positively correlated with the infiltration levels of 6 immune cells, including B cells, CD4+ T cells, CD8+ T cells, neutrophils, macrophages, and dendritic cells in LGG, PRAD, KIRC, COAD, and BRCA (Figure 7).

**Figure 8:**
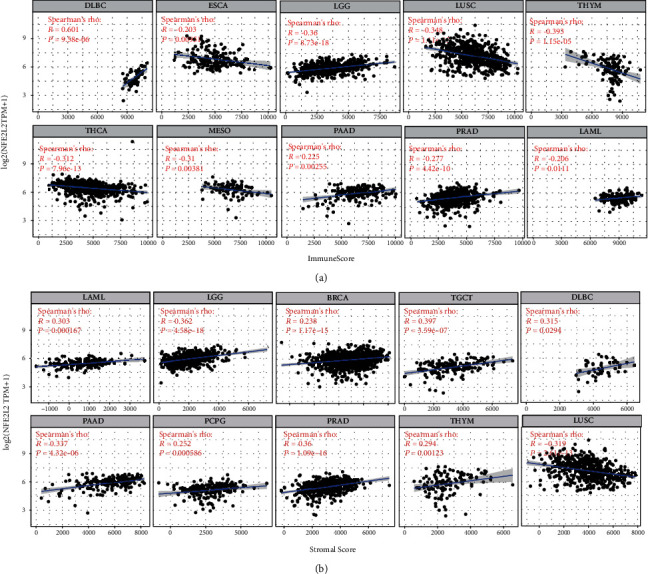
Correlation analysis between NFE2L2 expression and ImmuneScore/Stromal Score in cancers. (a) Correlation between NFE2L2 expression and ImmuneScore in DLBC, ESCA, LGG, LUSC, THYM, THCA, MESO, PAAD, PRAD, and LAML. (b) Correlation between NFE2L2 expression and StromalScore in LAML, LGG, BRCA, TGCT, DLBC, PAAD, PCPG, PRAD, THYM, and LUSC.

The immune score (i.e., ImmuneScore) and matrix score (i.e., StromalScore) were used to quantify the immune and matrix components in pan-cancer. NFE2L2 expression was positively correlated with the ImmuneScore in DLBC, LGG, PAAD, PRAD, LAML, and negatively correlated with the ImmuneScore in ESCA, LUSC, THYM, THCA, and MESO (Figure 8(a)). In addition, NFE2L2 expression was positively correlated with the StromalScore in LAML, LGG, BRCA, TGCT, DLBC, PAAD, PCPG, PRAD, and THYM and negatively correlated with the StromalScore in LUSC (Figure 8(b)).

**Figure 9:**
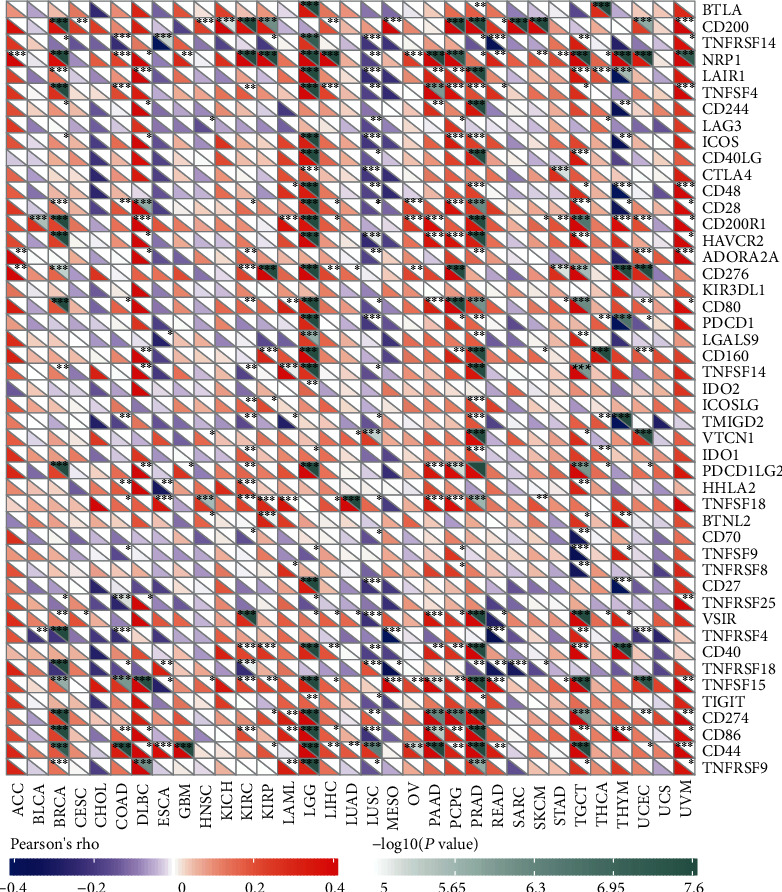
Correlation analysis of NFE2L2 expression levels with 40 common immune checkpoint gene levels in pan-cancer.

Immune checkpoint inhibitors (ICIs), as novel tumor immunotherapy agents, play an important role in tumor immunotherapy [29]. Subsequently, we analyzed the correlation between NFE2L2 expression and that of 40 common immune checkpoint genes. Interestingly, in LGG and PRAD, NFE2L2 expression was correlated with more than 30 immune checkpoint markers, such as TNFSF4, CD48, and CD28 (Figure 9). Collectively, these results strongly suggest that NFE2L2 plays a vital role in tumor immunity.

## 4. Discussion

Pan-cancer analysis can reveal similarities and differences in tumors, providing insights into cancer prevention and the design of therapeutic targets [30]. Recently, many studies have focused on pan-cancer analysis of the whole genome, revealing mutations, RNA alterations, and driver genes that are related to the occurrence and development of cancer, which is of importance for early diagnosis of cancer and development of biomarkers [31–35]. NFE2L2 is a transcription factor with alkaline lysine zipper structure, which plays a role in resisting oxidative stress and maintaining the body's redox homeostasis [36]. However, the roles of NFE2L2 in human pan-cancer have not been identified, and whether it can be used as a biomarker is still unknown. In the current study, we found that NFE2L2 is abnormally expressed in 22 cancer types and is significantly correlated with MMR gene mutation levels and DNA methylation. In addition, NFE2L2 expression was associated with poor prognosis (OS, DSS, and PFI) of patients, especially those with ACC, LGG, and PAAD. Furthermore, we observed that NFE2L2 expression was positively correlated with immune infiltration levels and the expression of immune checkpoint markers, especially in LGG. The above results strongly suggested that NFE2L2 may be used as a potential biomarker of LGG and play an indispensable role in tumor immunity.

Studies have shown that NFE2L2 could bind to KEAP1, which acts as a redox sensor to dissociate NFE2L2 from its cytoplasmic complex for translocation into the nucleus [37, 38]. In the nucleus, NFE2L2 binds to the antioxidant response element (ARE) to activate the expression of detoxification, antioxidant, and anti-inflammatory genes, establishing the NFE2L2/KEAP1/ARE signaling pathway [37]. Disrupting the balance of this pathway can lead to aging, inflammation, and tumor chemoresistance [39, 40]. In addition, several studies have indicated that NFE2L2 is upregulated in different types of cancers and correlates with tumor progression, aggressiveness, and poor prognosis [41]. Another study showed that cytoplasmic NFE2L2 expression was associated with patients' poor prognosis, while the nuclear NFE2L2 expression was associated with a more favorable prognosis [42]. Moreover, NFE2L2 is abnormally overexpressed in lung cancer cell line A549 [43]. These previous findings indicate NFE2L2 may be abnormally expressed in various cancers and play important roles in cancer progression and patients' prognosis. In this study, we found for the first time that abnormal expression of NFE2L2 exists in human pan-cancer including ACC, LGG, and PAAD. Survival analysis showed NFE2L2 expression was associated with poor prognosis in multiple cancers, especially in ACC, LGG, and PAAD. These results strongly indicate NFE2L2 is a potential prognostic biomarker in ACC, LGG, and PAAD.

Under normal conditions, MMRs ensures the stability of DNA replication. MMRs consists of multiple heterodimers, including MLH1/PMS2, MSH2/MSH6, and EPCAM, which can identify and correct gene mutations including base substitutions, insertions, deletions, or mismatches during DNA replication [44]. Mutations or defects in the MMR gene can lead to the accumulation of genetic errors, resulting in genomic or microsatellite instability, which contribute to the occurrence of tumors [45]. These indicate MMR gene mutation is a predictor of tumorigenesis. In this study, through correlation analysis, we found NFE2L2 expression was closely associated with the mutation levels of 5 MMR genes (MLH1, MSH2, MSH6, PMS2, and EPCAM) in human pan-cancer. In addition, alterations in DNA methylation status contribute to the development of cancer [46]. Recent research has shown that hypermethylation of the gene promoter is a common epigenetic feature of cancer [47, 48]. In our study, we also found that NFE2L2 expression was closely correlated with that of 4 DNA methyltransferases (DNMT1, DNMT2, DNMT3A, and DNMT3B) in human cancers, especially in COAD, KIRP, LGG, and UVM. These results strongly support our conclusion that abnormal expression of NFE2L2 may play an important role in tumorigenesis by regulating MMR gene mutation levels and DNA methylation.

The TME has been a recent focus of tumor research. The immune microenvironment composed of tumor-infiltrating lymphocytes (TILs; B cells and T cells) and other immune cells (dendritic cells, neutrophils, and macrophages) is an important part of the TME [49, 50]. Studies have shown that immune cells play an indispensable role as a double-edged sword in tumors to promote or inhibit tumor progression [51–53]. Under normal conditions, immune cells play an antitumor role by monitoring and destroying cancer cells [54]. On the other hand, studies have shown that cancer cells can evade the surveillance of immune cells through a variety of mechanisms [55–58]. TILs have been shown to be an independent predictor of patients' prognosis in cancers [59]. CD4+ and CD8+ T cells are crucial members of the TME that participate in specific antitumor immune responses [60]. Neutrophils secrete MMP9 into the TME, which contributes to angiogenesis, tumor progression, and metastasis in mouse transplantation models [61]. Macrophages are the first line of defense against tumor immunity. Instead of killing tumor cells, TAMs mediate tumor development [62]. These observations indicate that TILs play a crucial part in tumor progression. However, there are few studies about the roles of NFE2L2 in the immune microenvironment. In this study, we found that NFE2L2 expression was significantly correlated with the levels of 6 types of infiltrating immune cells (B cells, CD4+ T cells, CD8+ T cells, dendritic cells, macrophages, and neutrophils) in BRCA, COAD, KIRC, LGG, and PRAD. These results indicate that NFE2L2 may lead to tumorigenesis or inhibit tumor progression by changing the TIL status. These novel findings constitute substantial progress in identifying the important role of NFE2L2 in immune infiltration.

Immune scoring is an approach to evaluate the infiltrating CD3+/CD45RO+, CD3+/CD8+, or CD8+/CD45RO+ lymphocyte population at the center and edges of a tumor [63]. In the TME, a higher ImmuneScore or StromalScore indicates a larger number of immune or matrix components [64]. Our results revealed that NFE2L2 expression was positively correlated with the ImmuneScore in DLBC, LGG, LAML, PAAD, and PRAD and negatively correlated with the ImmuneScore in ESCA, LUSC, THYM, THCA, and MESO. In addition, NFE2L2 expression was positively correlated with the StromalScore in BRCA, DLBC, LAML, LGG, PAAD, PCPG, PRAD, TGCT, and THYM and negatively correlated with the StromalScore in LUSC. Moreover, the correlation between NFE2L2 expression and immune checkpoint markers implies the role of NFE2L2 in regulating tumor immunology in cancers, especially in LGG. These results further strongly indicate NFE2L2's important roles in tumor immunity.

## 5. Conclusions

In conclusion, the results of the present study indicated that NFE2L2 overexpression correlates with poor prognosis of patients and increases the infiltration levels of B cells, CD8+ T cells, CD4+ T cells, macrophages, neutrophils, and dendritic cells in many cancers, especially in LGG. In addition, NFE2L2 expression was found to be significantly correlated with the expression of immune checkpoint markers in LGG. Therefore, NFE2L2 may play a vital role in immune infiltration and be a potential prognostic biomarker for LGG.

## Data Availability

The data used to support the findings of this study are available from the corresponding author upon request.
